# Current state of cannabis use, policies, and research across sixteen countries: cross-country comparisons and international perspectives

**DOI:** 10.47626/2237-6089-2021-0263

**Published:** 2022-06-27

**Authors:** Ramdas Ransing, Pedro A de la Rosa, Victor Pereira-Sanchez, Jibril I. M. Handuleh, Stefan Jerotic, Anoop Krishna Gupta, Ruta Karaliuniene, Renato de Filippis, Eric Peyron, Ekin Sönmez Güngör, Said Boujraf, Anne Yee, Bita Vahdani, Sheikh Shoib, MJ Stowe, Florence Jaguga, Lisa Dannatt, Alexandre Kieslich da Silva, Paolo Grandinetti, Chonnakarn Jatchavala

**Affiliations:** 1 Department of Psychiatry BKL Walawalkar Rural Medical College Maharashtra India Department of Psychiatry, BKL Walawalkar Rural Medical College, Maharashtra, India.; 2 Instituto Cultura y Sociedad Universidad de Navarra Pamplona Spain Educación de la Afectividad y Sexualidad Humana, Instituto Cultura y Sociedad, Universidad de Navarra, Pamplona, Spain.; 3 Human Flourishing Program Institute for Quantitative Social Science Harvard University Cambridge MA USA Human Flourishing Program, Institute for Quantitative Social Science, Harvard University, Cambridge, MA, USA.; 4 Department of Child and Adolescent Psychiatry New York University Grossman School of Medicine New York NY USA Department of Child and Adolescent Psychiatry, New York University Grossman School of Medicine, New York, NY, USA.; 5 Saint Paul’s Hospital Millennium Medical College Addis Ababa Ethiopia Saint Paul’s Hospital Millennium Medical College, Addis Ababa, Ethiopia.; 6 Clinic for Psychiatry Clinical Centre of Serbia Belgrade Serbia Clinic for Psychiatry, Clinical Centre of Serbia, Belgrade, Serbia.; 7 Department of Psychiatry National Medical College Birgunj Parsa Nepal Department of Psychiatry, National Medical College, Birgunj, Parsa, Nepal.; 8 Elblandklinikum Radebeul Clinic for Psychiatry and Psychotherapy Medical Faculty Carl Gustav Carus Technical University Dresden Dresden Germany Elblandklinikum Radebeul Clinic for Psychiatry and Psychotherapy, Medical Faculty Carl Gustav Carus, Technical University Dresden, Dresden, Germany.; 9 Department of Health Sciences University Magna Graecia of Catanzaro Viale Europa Catanzaro Italy Psychiatry Unit, Department of Health Sciences, University Magna Graecia of Catanzaro, Viale Europa, Catanzaro, Italy.; 10 Clinique Belmont Genève Switzerland Clinique Belmont, Genève, Switzerland.; 11 Department of Psychiatry University of Health Sciences Erenköy Mental Health and Neurological Diseases Training and Research Hospital Istanbul Turkey Department of Psychiatry, University of Health Sciences, Erenköy Mental Health and Neurological Diseases Training and Research Hospital, Istanbul, Turkey.; 12 Clinical Neurosciences Laboratory Sidi Mohamed ben Abdellah University Fez Morocco Clinical Neurosciences Laboratory, Sidi Mohamed ben Abdellah University, Fez, Morocco.; 13 Department of Psychological Medicine University Malaya Centre of Addiction Sciences Malaysia Department of Psychological Medicine, University Malaya Centre of Addiction Sciences, Faculty of Medicine, University of Malaya, Kuala Lumpur, Malaysia.; Faculty of Medicine University of Malaya Kuala Lumpur Malaysia; 14 Qazvin University of Medical Sciences Qazvin Iran Qazvin University of Medical Sciences Qazvin, Iran.; 15 Department of Psychology University of Tehran Tehran Iran Department of Psychology, University of Tehran, Tehran, Iran.; 16 Department of Psychiatry Jawahar Lal Nehru Memorial Hospital Rainawari Kashmir India Department of Psychiatry, Jawahar Lal Nehru Memorial Hospital, Rainawari, Kashmir, India.; 17 School of Medicine Faculty of Health Sciences University of Pretoria Pretoria South Africa Department of Family Medicine, School of Medicine, Faculty of Health Sciences, University of Pretoria, Pretoria, South Africa.; 18 Moi Teaching & Referral Hospital Eldoret Kenya Moi Teaching & Referral Hospital, Eldoret, Kenya.; 19 Department of Psychiatry and Mental Health University of Cape Town Cape Town South Africa Department of Psychiatry and Mental Health, University of Cape Town, Cape Town, South Africa.; 20 Faculty of Medicine University of Vale do Taquari Lajeado RS Brazil Faculty of Medicine, University of Vale do Taquari, Lajeado, RS, Brazil.; 21 Addictions Service Department of Territorial Services ASL Teramo Teramo Italy Addictions Service, Department of Territorial Services, ASL Teramo, Teramo, Italy.; 22 Department of Psychiatry Faculty of Medicine Prince of Songkla University Songkla Thailand Department of Psychiatry, Faculty of Medicine, Prince of Songkla University, Songkla, Thailand.

**Keywords:** Cannabis, policies, legalization, global health, research

## Abstract

**Introduction:**

Varying public views on cannabis use across countries may explain the variation in the prevalence of use, policies, and research in individual countries, and global regulation of cannabis. This paper aims to describe the current state of cannabis use, policies, and research across sixteen countries.

**Methods:**

PubMed and Google Scholar were searched for studies published from 2010 to 2020. Searches were conducted using the relevant country of interest as a search term (e.g., “Iran”), as well as relevant predefined keywords such as “cannabis,” “marijuana,” “hashish,” “bhang “dual diagnosis,” “use,” “addiction,” “prevalence,” “co-morbidity,” “substance use disorder,” “legalization” or “policy” (in English and non-English languages). These keywords were used in multiple combinations to create the search string for studies’ titles and abstracts. Official websites of respective governments and international organizations were also searched in English and non-English languages (using countries national languages) to identify the current state of cannabis use, policies, and research in each of those countries.

**Results:**

The main findings were inconsistent and heterogeneous reporting of cannabis use, variation in policies (e.g., legalization), and variation in intervention strategies across the countries reviewed. European countries dominate the cannabis research output indexed on PubMed, in contrast to Asian countries (Thailand, Malaysia, India, Iran, and Nepal).

**Conclusions:**

Although global cannabis regulation is ongoing, the existing heterogeneities across countries in terms of policies and epidemiology can increase the burden of cannabis use disorders disproportionately and unpredictably. There is an urgent need to develop global strategies to address these cross-country barriers to improve early detection, prevention, and interventions for cannabis use and related disorders.

## Introduction

Cannabis is one of the most frequently used recreational psychoactive substances globally with an estimated 192 million users of cannabis in 2018,^1,2^ corresponding to 3.9% of the world population aged 15-64 years.^3^ Cannabis use is much more common in North America and high-income countries in Europe and Oceania than in low and middle-income countries (LMICs), where it has been increasing (only remaining low in Asia).^4^ Despite growing public support for its use in many countries, this substance is known to be associated with risk of mental health conditions, including suicidality, depression,^5^ and psychosis.^6^ Cannabis use has also been linked to adverse functional outcomes (e.g., aggression and school dropout) and disability, and to high direct and indirect socioeconomic costs.^7-9^

Cannabis dependence or problematic use is often influenced by sociopolitical environments, religion, culture, clinical practice, and policies and programs across countries.^10,11^ Most culturally distinct groups have used cannabis and other psychoactive substances throughout the ages, and they have accepted cannabis use as an established code of behavior.^12^ Moreover, acculturation has been associated with increased use of cannabis use.^13,14^ Understanding the epidemiology of cannabis use or dependence, policy measures, and research across countries is valuable to quantify the global extent of cannabis use and changes over time as well as to assist lawmakers, governments, and funding bodies in their decision-making regarding services and policies.

Nevertheless, few organizations regularly compile epidemiological data.^15-18^ Limited information about the various current legalizations, national harm reduction strategies, research trends, programs, and prevalence of cannabis use or dependence is available.^19^ This hampers the development of global strategies to understand the extent and impact of cannabis use and address problems that result. Nowadays, many countries and country regions are advancing with or considering legalization and there is little evidence on which to base assessments and foresee the impact of these challenges.^20,21^ Therefore, it is crucial to collate this information to obtain a global understanding of cannabis use and dependence and interventions to address them, highlighting critical gaps in these domains to enable better collaborative efforts and progress evaluations within the framework of the Sustainable Development Goals.^22^

Moreover, building capacities capable of framing and accompanying any open and extensive legal use of cannabis is necessary and must be considered. Strategies in this regard should emphasize both legal and medical frames. This might involve multiple stakeholders such as psychiatrists, general practitioners, pharmacists, etc. Against this background, we conducted a narrative review with the following aims: firstly, to describe the epidemiology of cannabis use or dependence, legalizations, and any existing harm reduction strategies (i.e., policies, programs, and practices) in different countries across the world; and secondly, to describe current trends of cannabis-related research in these countries.

## Material and methods

### Team setup

The first author (RR) recruited team members by addressing an invitation to members of the Early Career Psychiatrists (ECP) Section of the World Psychiatric Association (WPA) and the Network of Early Career Professionals working in the area of Addiction Medicine (NECPAM). Sixteen people from different countries (n = 16) accepted the invitation and contributed to all the stages of the study (another four colleagues accepted the initial invitation but were unable to perform all the required tasks). These sixteen participants contributed with data from their countries, which we grouped by WPA geographical divisions (regions and zones).

### Data collection

The narrative review and critical analysis of available literature were conducted as per protocol.^23,24^ The first author (RR) requested all country representatives to conduct independent searches of literature from their respective countries. Online databases (PubMed, Google Scholar) were searched for peer-reviewed articles (including case reports and letters to editors) published from January 2010 to December 2020; a time frame that would provide a decade-long perspective. Searches were conducted using the relevant country of interest as a search term (e.g., “Iran”), as well as relevant keywords such as “cannabis,” “marijuana,” “hashish,” “bhang,” “dual diagnosis,” “use,” “addiction,” “prevalence,” “co-morbidity,” “substance use disorder,” “legalization,” “policy,” etc. These key terms were used in multiple combinations to create strings to search study records’ titles and abstracts. Country representatives also searched official policy documents, statements, and websites from their governments. Data from the World Health Organization (WHO) and United Nations Office on Drugs and Crime (UNODC) were also considered. National peer-reviewed general medical or psychiatric journals were searched manually. Results that did not pertain to cannabis use or dependence and those focused on biotechnological aspects of the tetrahydrocannabinol (THC) or cannabidiole (CBD) molecules were excluded.

Three authors (RR, PAR, and CJ) clarified some ambiguous terms such as decriminalization and legalizations. Group discussions were held via online messaging and conferencing platforms. Subsequently, the two authors not involved in data collection (RR and VP-S) compiled and summarized the data retrieved, seeking clarifications when needed; the information collected from participant countries was summarized and tabulated under the following domain headings: epidemiology, legislation, harm reduction strategies, and research areas. Three authors (RR, VP-S, and PG) who had not taken part in the literature search critically analyzed the data.

## Results

### Epidemiology

We found a wide range of variations in terms of epidemiological aspects of cannabis use/dependence across the included countries (Table 1). A higher prevalence of cannabis use or dependence was found among teenagers or younger adults than among elderly adults in European (Italy, Spain), African (South Africa, Kenya, Ethiopia),^17^ and Asian countries (Nepal, Iran). In comparison, some Asian countries (Thailand) have reported that the number of cannabis users is shrinking. In the literature reviewed, cannabis use has often been associated with aggressive behavior, early onset of schizophrenia, and comorbid use of other substances such as opioids (Iran)^25^ and alcohol (Ethiopia).^26^ It has also been strongly associated with mood and anxiety disorders,^27^ truancy,^28^ school dropouts, unemployment, other drug use, and risky sexual practices.^29^ The prevalence of cannabis use seems higher among males and those with a family history of cannabis dependence and poor peer support.^28^ In most countries (e.g., Germany), cannabis is the third most common substance use disorder after alcohol and amphetamines.^30^ Furthermore, researchers worldwide (India, Nepal) have attempted to determine the relationship between cannabis use and psychotic, mood, or anxiety disorders and comorbidities with substance use disorders.^31-33^ Variations were also observed in terms of the patterns of cannabis use in national surveys when conducted (e.g., annual, last 3/6 months, daily, last month, or lifetime prevalence^15,34^).

**Table 1:** Epidemiological characteristics of CU/CD across countries, grouped by World Psychiatric Association regions

Region/zone/country	Epidemiology		
	General population	Adolescent population	Additional information
Asia (n = 5)			
Southern Asia			
India	Age: 18-75 years CU (lifetime): 3.3%^34^ CD: 0.25%^34^	Age: 10-17 years CU (lifetime): 0.9%^34^	Clinical population: 11.6%^34^
Nepal	Age: 15-64 years CU (last 12 month): 3.2%^18^	NA	Medical students CU (NA): 12.8-18.5%^35^^,^^36^
Thailand	Age: 12-65 years CU (lifetime): 5.05% CU (last 12 months): 0.2%^37^	Age: 15-24 years CU (last 3 month): 2.1%^38^	Clinical population Age: 18-60 years CD (last 3 months): 6.5%^29^
Malaysia	NA	Age:12-15 years CU (lifetime): 1.5% CU (last 30 days): 1.2%^2^ Age: 13-17 years CU (lifetime): 4.4%^28^	Drug users CU (last 12 months); 3%^39^
Central and Eastern Asia			
Iran	Age: 15-64 years CU (last 12 months): 0.56%^40^	High school students Age: 15-18 years CU (lifetime): 5%^25^	Young people Age: 15 to 29 years CU (lifetime): 4%^41^ College students Age: 19-23 years CU (lifetime): 2%^25^
Europe (n = 6)			
Central Europe			
Serbia	Age: 15-64 years CU (lifetime): 7.7% CU (last 12 months):1.6%^42^	Age: 14-18 years CU (last 12 months): 5%^16^	Cannabis use (0.5%) among the adult population.^42^^,^^43^
Germany	Age: 18-64 years CU (lifetime): 1.2%^43^ CU (last 12 months): 6.9%^44^	Age: 14-18 years CU (lifetime): 10%^44^ CU (last 12 months): 8%^44^ CU (last 30 days): 2.9%^44^ CD: 3.9%^44^ Age: 12-13 years CU (last 12 months): 1.9%^44^	Younger adult Age: 18-34 years CU (lifetime): 13.3%^45^
Southern Europe			
Turkey	Age: 15-64 years CU (lifetime): 2.7% CU (last 12 months): 1.1%^46^ CU (last 30 days): 0.8%^47^^,^^48^	Age: 14-18 years CU (last 30 days): 2.3%^49^	Young adults Age: 18-34 years CU (last 12 months): 1.8%^43^
Spain	Age: 15-64 years CU (lifetime): 35.2%^43^ CU (last 12 months): 11% CU (last 30 days): 9.1%^43^ CD (CASTS): 1.6%^50^	Age: 14-18 years CU (lifetime): 33%^50^ CU (last 12 months): 27.5%^50^ CU (last 30 days): 19.3%^50^ CD (CASTS): 2.3%^50^	In 2018, Cannabis (38.5%) was found to be the second-most frequently reported substance used during first admission for any substance use treatment, after cocaine.^50^
Italy	Age: 15-64 years^43^ CU (lifetime): 32% CU (last 12 months): 14.3% CU (last 30 days): 6.9%	Age: 15-24 years^43^ CU (lifetime): 34.2% CU (last 12 months): 22.3% CU (last 30 days): 11%	Young adults Age: 15-34 years^43^ CU (lifetime): 37.5% CU (last 12 months): 20.3% CU (last 30 days): 9.9%
Western Europe			
France	Age: 18-64 years CU (last 12 months): 11%	Age: 15-16 years CU (lifetime): 31% CU (last 30 days): 17%^51^	Cannabis experimentation: 32.8%^52^
Americas (n = 1)			
South America			
Brazil	Age: 12-65 years CU (lifetime): 7.7% Age:15-64 years CD (last 12 months): 2.5%^53^	Age: 14-17 years CU (lifetime): 4.3% CU (last 12 months): 3.4%^54^	College students (Age: 18-35 years CU (lifetime): 26.1% CU (last 12 months): 13.8% CU (last 30 days): 9.1%^54^ Street children (Age: 10-18 years) CU (lifetime): 40.4% CU (last 12 months): 32.1% CU (last 30 days): 25.4%^55^
Africa and the middle west (n = 4)			
Northern Africa			
Morocco	Age: 15-64 years CU (lifetime): 5% CU (last 12 months): 3.94%^46^	Age: < 18 years CU (lifetime): 4.1% CU (last 12 months): 3.1% CU (last 30 days): 2%^19^^,^^46^	Female Age: 15-17 years CU (lifetime): 2.1% CU (last 12 months): 0.7% CU (last 30 days): 0.6%
Eastern and Southern Africa			
South Africa	Age: 15-64 years CU (lifetime) 10.8%^56^ CU (last 12 months): 3.65% CU (last 3 months): 4%^57^	No country-wide epidemiological data for age group (12-18 years)^17^	Cannabis is most common primary drug used among the people in the age group (< 20 years).^58^
Kenya	Age: 15-65 years CU (lifetime): 4.5%^46^ CD: 1.2%	Adolescents and college students CU (lifetime): 1.7-8.1%^59^^,^^60^	Household heads CU (lifetime): 0.6%^61^ Inpatient rehabilitation CU (lifetime): 64%^62^
Ethiopia	Age: not specified CU (lifetime): 42.2%^26^^,^^46^ CU (last 12 months): 11%^63^	NA	Prison population CU (lifetime): 3.6%^64^

CASTS = Cannabis Abuse Screening Test Scale; CD = cannabis dependence; CU = cannabis use; NA = not available.

### Legalizations and decriminalization

The process of lifting prohibitions against cannabis use is known as legalization, while sparing criminal sanctions (such as fines, prison, or mandated treatment) against people possessing or using it is known as decriminalization.^65^ Cannabis consumption is legally prohibited in most countries. Country-specific details on these prohibitions and decriminalization laws are listed in Table 2. Almost all countries have adopted legal prohibitions as one of the core strategies to reduce cannabis use. Legal prohibitions seem to have substantially reduced cannabis use in many countries (e.g., before any legal prohibitions, Kathmandu was considered a ‘hippie hub’ inviting tourists and promoting hashish and tourism eventually). Some countries have harsh policies (e.g., Malaysia), while others are lenient (e.g., Spain). In Spain, article 368 of the Penal Code distinguishes between drugs that cause and do not cause serious health damage. Given that drug-induced harm is related to drug quantity, a person may possess up to 100 grams of cannabis for personal consumption.^66^ Most of the countries in our review have prevalent positive social attitudes towards the future legalization of cannabis (Table 2). However, political and religious factors are affecting the implementation of cannabis legalization in almost all countries.^67,68^

**Table 2:** Current status of cannabis-related prohibitions, decriminalization, and legalizations across countries

Country	Decriminalized	Legalized for use other than medicinal use	Private (home based) cannabis production/cultivation	Permitted for medicinal use and research purpose	Prohibition of cannabis use and additional points
India	No	No	No	Yes, commonly used in Indian systems of medicine (Ayurveda, Siddha, and Unani)	Prohibition: cultivation, possession, trafficking, and consumption of all cannabis preparations except bhang (with a maximum threshold of the narcotic principle [THC] set between 0.2-0.5%).^69^
Nepal	No	No	No	Not permitted	Prohibition: cultivation, possession, trafficking, and consumption of all cannabis preparations except bhang.^70^ Punishment method: monetary fine and imprisonment, No harsh punishment.^69^
Thailand	Yes	Yes	Yes (2020)^71^	Yes (2018), for medical conditions such as cancer, Parkinson’s disease, demyelinating disorders, epilepsy^72^	Prohibition: trafficking.^73^
Malaysia	No	No	No	No	Prohibition: cultivation, possession, trafficking, and consumption of all cannabis preparation.^74^
Iran	No^75^	No^75^	No	No, use of dronabinol capsules and Sativex^®^ sprays for some limited research projects	Prohibition: cultivation, possession, trafficking, and consumption of all cannabis preparation.^76^
Serbia	No	No	No	No	Prohibition: cultivation, possession, trafficking, and consumption of all cannabis preparation.^77^^,^^78^
Germany	No	No	No	Yes^79^	Prohibition: possession, trafficking, and purchase of recreational cannabis.^80^^,^^81^ 2011: permitted for medicinal cannabis products^81^; 2017: permitted for seriously ill patients with no therapeutic alternative.^81^
Turkey	No	No	No	Yes, Sativex oromucosal spray for medical conditions^82^	Prohibition: possession, trafficking, sale, and purchase of recreational cannabis.^83^
Spain	Yes (only for personal use^66^)	No, Penal Code Law, Nº 368^84^	Yes (only for personal use).	No. In 2010, Sativex^®^ was approved for treating spasticity symptoms of multiple sclerosis resistant to other drugs.^66^	Prohibition: production and trafficking.^84^ Cannabis clubs claim to help patients to obtain cannabis for medicinal use. Personal possession of up to 300 grams is permitted for medicinal or recreational purposes (intended use of 10 grams/day for up to 30 days). Self-cultivation is allowed in a quantity of up to six female plants per person, up to a limit of five persons, with authorization for storage corresponding to the annual harvest.^66^
Italy	Yes	No	No (a new law is currently under discussion in parliament)	Yes, for medical conditions such as chronic pain, multiple sclerosis, spinal cord injury, nausea and vomiting caused by chemotherapy, radiotherapy	Prohibition: trafficking, and selling cannabis (even free of charge).^85^ Possession for personal use is permitted (with a maximum threshold of the narcotic principle [THC] set between 0.2-0.5%.
France	No	No	No	Yes, only for medical indications since October 2020	Prohibition: possession, production, trafficking, and distribution.^84^
Brazil	No	No	Pernambuco state so far^86^	Yes, for medicinal use such as palliative care without other therapeutic alternatives, refractory epilepsy, multiple sclerosis	Prohibition: possession, production, and distribution other than personal and private use.^87^^,^^88^
Morocco	Under consideration (in parliament)	Under consideration (in parliament)	No	Not for medicinal use; permitted for research.	Harvesting of cannabis for medicinal and industrial use is permitted.
South Africa	Yes (to be ratified by parliament)	No	Yes^7^	No for medicinal use; permitted for research.^7^	Prohibition: Cultivation, possession, and trafficking.
Kenya	No	No	No	No	Prohibition: cultivation, possession, and trafficking.^89^ Marijuana Control Bill, 2018 (in parliament) seeks to legalize cannabis for medical and recreational purposes.^90^
Ethiopia	No	No	No	No	Prohibition: cultivation, possession, trafficking, and consumption of all cannabis preparations. But there is no policy or law.

Punishments for violations of legal regulations include imprisonment and fines across all countries.

### Harm reduction strategies

Table 3 lists national and local level harm reduction strategies adopted in the countries represented by our team. Efforts in training and education of service users, service providers, the general public, youth, and adolescents are currently being carried out in all of these countries. Simultaneously, school-based programs are being run in some countries, such as Nepal, France, and Spain, while in other countries like India^91^ they are yet to be widely implemented. Some of these programs, such as “Unplugged,”^92^ have been implemented in several countries with excellent results, but not all schools can afford the expense. One initiative in Spain to raise awareness about substance abuse among schoolers and university students is based on short film contests.^93^ Awareness programs targeting the general population were found across the countries represented by our team. The Malaysian government has initiated two programs, “Sayangi Hidup, Elak Derita Selamanya” (SHIELDS) and “Tomorrow’s Leader,” which are aimed at providing drug education and prevention in educational institutes. In Malaysia, compulsory classroom-based antidrug education programs are being delivered in secondary schools. Cannabis is depicted negatively in the media to increase risk awareness (France, Spain, Kenya, and Iran).^94^ Rehabilitation services along with outpatient and inpatient services supervised by psychiatrists are the mainstay treatment for patients with cannabis disorders in all countries.^29^ Some high-income countries harness digital tools to provide support programs.^95^

**Table 3:** Preventive and therapeutic strategies for cannabis use across countries

Country	Commonly used preventive and therapeutic strategies or programs for cannabis use
India	Rehabilitation programs for drugs including cannabis, integrated rehabilitation centers for addicts (MoSJE),^96^ dedicated centers
Nepal	Rehabilitation programs, education
Thailand	Matrix model of outpatient stimulant abuse treatment,^97^ community-based recovery models, rehabilitation programs
Malaysia	Rehabilitation programs, holistic health recovery program in the criminal justice system,^98^ school-based programs for early detection, community-based clinics (e.g., cure and care service centers [CCSC] run by national anti-drugs agency), substance clinic at government hospitals, private rehabilitation centers
Iran	Matrix model, relapse prevention services, brief interventions at outpatient treatment centers for substance use disorders, school-based programs for early detection, life skills training programs in schools, social media-based approaches (e.g., educational short films, clips)
Serbia	Government action plan for suppressing abuse of drugs for the period 2014-2021,^99^ a multi-country regional project (government and activists), dedicated centers
Germany	Cooperation between insurance providers, the government, non-governmental institutions, policy measures reducing the availability of illicit drugs, school-based prevention activities (e.g., life skills, critical thinking about drug use), family oriented prevention programs (e.g., parenting skills, protective role), outpatient treatment centers serving as additional contact points, harm reduction interventions targeting migrants, rehabilitation programs
Turkey	Turkey’s national strategy and action to combat illegal drugs (2018-2023), prevention programs at several levels in coordination with the relevant organs, AMATEM, alcohol and substance addiction treatment centers, social norms approach for prevention in adolescents and young adults.^49^^,^^100^
Spain	The action plan on addictions establishes several prevention programs at different levels 1. Risk awareness raising through media 2. Universal school-based programs 3. School-based surveys for early detection 4. Rehabilitation programs 5. Market control through military and police forces Additionally, cannabis clubs claim they protect consumers from unlawful distribution and problematic use.
Italy	The new national action plan is logically divided into five main areas of intervention: 1. Prevention – early information, universal and selective prevention, early detection of use of drugs (early detection), and educational approach; 2. Treatment and diagnosis of drug addiction – early contact, prompt reception, diagnosis, and appropriate therapies and contextual prevention of related diseases; 3. Rehabilitation and reintegration – social and work; 4. Monitoring and evaluation; 5. Legislation, law enforcement, and juvenile justice – both on the ground and on the internet. The five areas indicated are grouped into two large containers: 1. Demand reduction: prevention, treatment and diagnosis, rehabilitation, and reintegration; 2. Reduction of supply: monitoring and evaluation, legislation, law enforcement, and juvenile justice.
France	Special follow-up for young people, motivational therapy, rehabilitation programs^101^
Brazil	Specific psychosocial attention centers, rehabilitation program, dedicated centers
Morocco	Limiting the area harvested and limiting production, presenting alternatives for cannabis farmers, media sensitization, and school education, extending and increasing addiction centers, replacement therapy (methadone)
South Africa	School or youth-based programs, The South African National Council on Alcoholism and Drug Dependence (SANCA) runs seasonal campaigns to raise risk awareness through media.
Kenya	Public education through broadcast and print media,^102^ primary and secondary school curricula –substance use education modules, life skills training program for primary school children, inpatient rehabilitation
Ethiopia	Motivational therapy is the most commonly used psychotherapy for cannabis use in Ethiopia. Cognitive behavioral therapy is also used in dual treatment with comorbid psychiatric disorders in Ethiopia

We identified these barriers to accessing health care services: social stigma (Nepal, Iran, Malaysia), lack of acceptance by religious traditions (Nepal, India), “myths” (e.g., misinformation in Kenya stating that cannabis gives physical and sexual strength, or ideas that cannabis is harmless or makes its users more intelligent, etc.).

### “Medical cannabis”

Some components of cannabis are approved and legal for medical use in some countries (Table 2). Additionally, some countries (Thailand) have permitted household cannabis cultivation. Many countries have already permitted cannabis for research purposes, so it is currently being used for many conditions such as an appetite stimulant for cachexia and anorexia, loss of appetite in cancer patients or in patients who have acquired immunodeficiency syndrome (AIDS), and in anorexia nervosa; and in glaucoma, targeting a hypotensive effect.

### Cannabis use/dependence research

**Figure 1:**
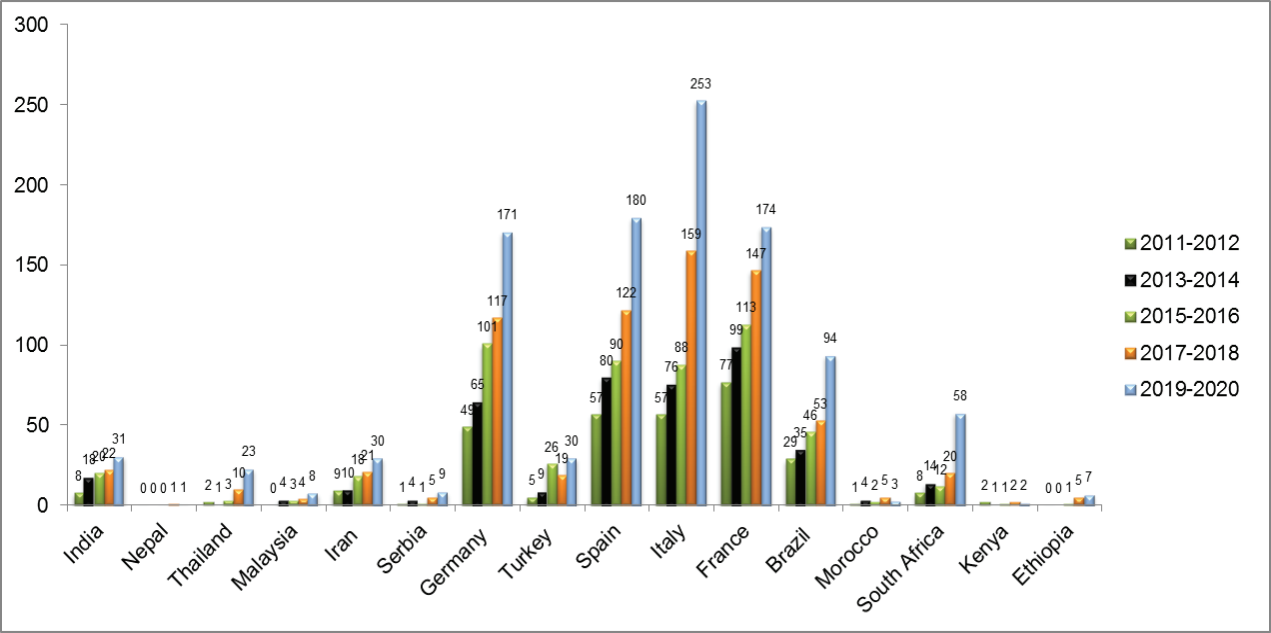
Trends of publication of articles about cannabis use or dependence indexed on the PubMed database over time (2010 to 2020)

Figure 1 depicts the trend of publication of cannabis-related articles indexed on the PubMed database over time (2010 to 2020). Over this decade, cannabis research was disproportionately dominated by European countries (Italy, Spain, Germany, and France). In most of the Asian and African countries included in our sample, researchers have mainly focused on cross-sectional (India, Nepal) and retrospective chart reviews (India), and there are only a few prospective studies (Table 4). Systematic-reviews, meta-analyses, cross-sectional, prevalence, and comorbidity studies, government reports, census reports at rehabilitation centers, and single-center studies at medical institutions were all identified in the literature reviewed. Large-scale general population studies are lacking due to inadequate funds and stringent policies in Asian countries. Studies of the benefits of cannabidiol as measured by electroencephalography (EEG) signals and genetic diversity studies were recently conducted in Morocco, France, Italy, and Iran.

**Table 4:** Cannabis use/dependence research across countries

Countries	Research domains and designs	Challenges for conducting research
India	Cross-sectional survey (co-morbidities, national survey), limited longitudinal studies	Legalization of cannabis, most Indian studies are on co-morbidities, inadequate funds for interventional studies
Nepal	Cross-sectional, prevalence studies, government reports, census report at rehabilitation centers, and single-center studies at medical colleges	Inadequate funds for interventional studies or large-scale general population study
Thailand	Cross-sectional, prevalence studies, government reports	Inadequate funds for interventional studies or large-scale general population study
Malaysia	Restricted	Harsh drug policy, motivation of researchers, clinicians, and governments
Iran	Cross-sectional studies, epidemiological studies, national survey, systematic review, meta-analyses, co-morbidity survey, service utilization, chemistry, pharmacological and toxicology, efficacy of cannabidiol treatment for anxiety, fear, and PTSD	Harsh legal policies, motivation of researchers, clinicians, and governments
Serbia	National surveys, epidemiological studies, community cross-sectional studies on attitudes, and knowledge about medical aspects of cannabis	Motivation of researchers, clinicians, and governments
Germany	Comorbidities,^103^ a prospective longitudinal community study (causal relationship),^104^ small-scale controlled experiment^105^	The new GDPR has been affecting epidemiological research since 2016^63^
Turkey	Nationwide surveys (annually by governmental authorities, such as the Turkish National Monitoring Centre for Drugs and Drug Addiction [TUBIM]),^47^ co-morbidity survey^48^	Motivation of researchers, clinicians, and government
Spain	National surveys and university or clinical research (mainly cross-sectional or cohorts), qualitative research.	The new GDPR has been affecting epidemiological research since 2016^63^
Italy	Cross-sectional studies,^106^ epidemiological studies,^107^ surveys,^108^ systematic review,^109^ meta-analyses,^110^ co-morbidity studies,^111^ pharmacological and toxicology,^112^ efficacy of cannabidiol treatment for anxiety,^113^ and use in PTSD^114^	The new GDPR has been affecting epidemiological research since 2016^63^
France	Epidemiological studies (prevalence and correlates)	Inadequate funds and human resources for interventional studies or large-scale general population study^115^
Brazil	Epidemiological studies,^116^ national surveys, medical application and use^117^	Legalization of cannabis, motivation of researchers, clinicians, and government

GDPR =European General Data Protection Regulation; PTSD = post-traumatic stress disorder.

## Discussion

Cannabis use/dependence seems far more prevalent in some countries or regions compared to others. However, it is far less common than alcohol, tobacco, or opioids in many countries. Among those countries (Morocco, Nepal, and India), some possible reasons for the higher prevalence may be unemployment poverty, lack of harsh punishment,^118^ acceptance of cannabis as medicine, cultural or religious acceptance, and a favorable climate for cultivation. It has not been possible to challenge the deep-rooted acceptance and religious basis of cannabis consumption in the culture in some countries (India and Nepal), which may hamper harm reduction strategies globally or nationally.^10,118^ Furthermore, the surge in cannabis use or dependence in European countries could be due to low risk perception, cultural acceptance, and acculturation.^119^ Also, a higher prevalence of cannabis use was found among the adolescents with migration background (1.9%) compared to those without migration background (0.2%).^44^

Some African countries and other LMICs have limited or no data on cannabis use. Also, published literature suggests that the quality of epidemiological data is often poor in LMICs due to a lack of national surveys and research funding.^119,120^ Furthermore, the lack of homogenous data collection methods, periodicity, or standard definitions of cannabis use in surveys may affect international efforts to develop national or global cannabis prevention strategies or interventions. Overreliance on self-report of substance use, barriers to marginalized populations (e.g., ex-prisoners or homeless persons) being involved in research, and religious barriers often affect the quality of data collected in national or population-based surveys. While many countries have conducted national surveys, they lack information regarding clinical characteristics, comorbidities, and interventions. Also, since use of cannabis is banned in many countries, epidemiological research is mostly conducted in clinical populations. We observed that cannabis-related research is limited in terms of interventions or policies in high-income countries (due to low prevalence) and LMICs (due to lack of resources or financial and legal constraints).^120,121^ Regional efforts should gather detailed findings in clinical and non-clinical samples and on interventions and trends.

Cannabis use is commonly associated with being young, male gender, having lower levels of education, unemployment, adverse childhood events, being unmarried, and low socio-economic status in almost all studied countries. Further, vulnerable or marginalized populations such as female sex workers or “street boys” have a higher prevalence than the general population.^122,123^ Also, countries across world regions have reported an increase in the prevalence of cannabis compared to previous surveys.

Legal prohibition is the most commonly adopted measure against cannabis use across the countries reviewed. Malaysia was found to have the lowest prevalence of cannabis use or dependence among the countries studied. This could be due to potential underreporting because of harsh legal prohibition and punitive drug policies. Furthermore, this has affected cannabis-related research initiatives. Harsh policies also affect access to de-addiction services, research, and service development. The extent of public health interventions (such as awareness-raising campaigns, skills training) in these countries was deficient, probably leaving behind many patients and at-risk people. The Malaysian example suggests that punitive drug policy has failed to yield the expected benefits of reduction in cannabis use; therefore, countries like it are considering the decriminalization of possession of drugs for personal use.^124^ Punitive policies also seem to have led to negative social and health outcomes: higher drug use relapse, overcrowding of prisons and detention centers, potential outbreaks of infectious diseases, social stigma, unemployment, and an increase in socioeconomic distress.^124^

Countries with considerable prevalence of cannabis use/dependence have initiated awareness campaigns in collaboration with the media, psychological interventions, and educational programs in the school curricula. Some countries (e.g., Italy) have developed a national early warning system to counter the consumption and sale of cannabis online. In some Spanish regions, cannabis users have established associations known as “cannabis clubs” to protect themselves from black-market goods and detect problematic cannabis use. Still, many barriers (e.g., stigma, myths, religious perceptions, lack of planning or evidence-based interventions, and lack of trained professionals) jeopardize the success of such efforts.

Use of cannabis or related products for medicinal purposes (mainly THC and CBD) is allowed in many countries (Brazil, Thailand, India, Spain, Nepal, and Germany) for different medical conditions (e.g., pain in terminally ill patients, cancer, multiple sclerosis) and within different medical systems (Ayurveda, Unani, and Siddha traditions in India, and Nepal). There is a positive stance towards the legalization of ‘medical cannabis’ in many countries (Serbia, Malaysia).^124-126^ In some countries, specific regions have allowed household production of cannabis (e.g., the state of Pernambuco in Brazil). However, this may lead to an increase in the prevalence of cannabis use or dependence in the future, as observed in Germany. In 2020, Brazil’s National Health Surveillance Agency (Agência Nacional de Vigilância Sanitária [ANVISA]) approved regulation of the medical use of cannabis in Brazil. After this decision, cannabis-based products started to be sold in pharmacies all over Brazil. However, the decriminalization of cannabis possession for consumption and plant cultivation is still pending judgment by the Brazilian Supreme Court.^127^

The experiences with alcohol and tobacco in many countries have shown that marketing and distribution can be very difficult to control in commercially‐driven approaches to psychoactive substances and can be catastrophic for public health, even with well‐intentioned regulations.^20^ Cannabis legalization, even with market regulation, will increase cannabis use-related disorders. In the United States, cannabis use and dependence increased in states that legalized medical use with a high prevalence of cannabis use disorders and severe psychiatric disorders, in addition to automobile accidents. The cannabis legalization experiment in other countries simply repeated the histories of other substances and their impact on public health.^128^

Considering the future possibilities of cannabis legalizations, efforts should be made towards ensuring the existence of sufficient specialized medical workforce and health services across countries, creating awareness of harmful use and rigorous monitoring of dependence and awareness and prevention campaigns.^129^ Despite growing cannabis use and its potential risks, research in many countries is limited due to religious (India), political, cultural, economic, and political barriers (e.g., Malaysia, Iran). Cannabis researchers in many countries may struggle to obtain institutional support or funding for mental health-related research. We have not investigated trends in cannabis use or dependence, but most countries (e.g., Turkey, Italy) have reported an increasing trend as compared with previous studies.

### Strengths, limitations, and future directions

This narrative review has facilitated identification of knowledge gaps and the scope of existing literature through extensive searching of literature (both published and gray). The critical evaluation of literature by independent reviewers has reduced the potential for group-based-bias entering the conclusion compared to the consensus approach.^23^ This review’s limitations include use of a restricted number of databases (PubMed, Google Scholar), which were searched by only one reviewer per country, restriction criteria that may not have captured all information intended, and no analysis was conducted of the quality of papers included. The lack of closely matching criteria across the reviewed countries precluded us from conducting a systematic review.

Furthermore, independent reviewers and country-wise contributors’ unintentional bias due to divergent views about literature cannot be ruled out. Despite these limitations, this review with a critical approach is the first primary source of evidence. It is therefore valuable for development of global strategies for cannabis use disorders and harmonization of cannabis research worldwide. The study findings will be helpful precursors to future scoping, systematic reviews, and meta-analyses.

## Conclusion

Our cross-country literature review involving all WPA regions, eight zones, and 16 countries provides several critical directions for research in epidemiology, policy, clinical programs, research, and international collaboration related to cannabis. Several countries have cannabis control or prevention policies but inadequately prepared services for cannabis use/disorders. In many countries it is necessary to establish national surveillance systems to monitor the changes or patterns of cannabis use and focus on developing preventive, diagnostic, and rehabilitation strategies. There is also a need to develop comprehensive research and service strategies for individual countries and globally, blending evidence-based and culturally-sensitive perspectives to design effective public health policies.
